# Serine-Arginine Protein Kinase 1 (SRPK1): a systematic review of its multimodal role in oncogenesis

**DOI:** 10.1007/s11010-022-04456-7

**Published:** 2022-05-18

**Authors:** William P. Duggan, Emer O’Connell, Jochen H. M. Prehn, John P. Burke

**Affiliations:** 1 Department of Colorectal Surgery, Beaumont Hospital, Dublin 9, Ireland; 2 Department of Physiology and Medical Physics, Royal College of Surgeons in Ireland, Dublin 2, Ireland

**Keywords:** Serine-Arginine Protein Kinase 1 (SRPK1), Alternative splicing, Cancer, Prognosis, Chemotherapy resistance

## Abstract

Alternative splicing is implicated in each of the hallmarks of cancer, and is mechanised by various splicing factors. Serine-Arginine Protein Kinase 1 (SRPK1) is an enzyme which moderates the activity of splicing factors rich in serine/arginine domains. Here we review SRPK1’s relationship with various cancers by performing a systematic review of all relevant published data. Elevated SRPK1 expression correlates with advanced disease stage and poor survival in many epithelial derived cancers. Numerous pre-clinical studies investigating a host of different tumour types; have found increased SRPK1 expression to be associated with proliferation, invasion, migration and apoptosis in vitro as well as tumour growth, tumourigenicity and metastasis in vivo. Aberrant SRPK1 expression is implicated in various signalling pathways associated with oncogenesis, a number of which, such as the PI3K/AKT, NF-КB and TGF-Beta pathway, are implicated in multiple different cancers. SRPK1-targeting micro RNAs have been identified in a number of studies and shown to have an important role in regulating SRPK1 activity. SRPK1 expression is also closely related to the response of various tumours to platinum-based chemotherapeutic agents. Future clinical applications will likely focus on the role of SRPK1 as a biomarker of treatment resistance and the potential role of its inhibition.

## Introduction

Cancer is a heterogeneous entity characterised by at least six biological hallmarks. These hallmarks include; uncontrolled proliferation, replicative immortality, angiogenesis, invasion and metastasis, evasion of growth suppression and avoidance of cell death [1]. Oncogenesis is driven by a host of deregulated signalling pathways that allow cells move through various processes to acquire additional oncogenic properties [2]. Though all cancers may display similar characteristics, molecular differences within specific cancer subtypes are frequently observed, and can have a profound impact on disease progression, treatment response and survival [3, 4]. Apart from molecular differences, differences in tumour structure and environment are also important. Each tumour is made not only of cancerous cells, but an entire tumour microenvironment, within which exists stromal cells, immune cells and even bacteria in some instances [5, 6]. Disentanglement of the interplay that exists between individual tumours and their host microenvironment, will improve understanding of aberrant pathways that promote cancer progress and development [7]. Through identification and targeting of such pathways, new treatment options are emerging which will improve and help personalise future cancer treatments [8, 9].

Alternative splicing (AS) is one of the key drivers of protein diversity in humans. It describes the process by which introns and exons are added and removed in various combinations resulting in the production of various protein transcripts [10]. Interestingly splicing isoforms of a single pre-mRNA transcript can often have antagonistic functions, thus can enhance or suppress various metabolic processes [11]. ‘Hallmarks of cancer’ are frequently associated with a switch in splicing towards a more aggressive, invasive phenotype. For example the pro-angiogenic rather than the anti-angiogenic vascular epithelial growth factor A (VEGFA) isoform is known to predominate in numerous malignancies [11–14].

Serine/arginine protein kinase 1 (SRPK1) is an enzyme encoded by a gene located on chromosome 6 in humans. It is known to be overexpressed in normal pancreas and testicular germ cells and underexpressed in glia [15–17]. SRPK1 plays a critical role in regulating AS, via the phosphorylation of various splicing factors rich in serine/arginine domains (SR proteins) [18]. SRPK1 is structurally made up of two kinase domains that are separated by stretched divergent spacer sequences [19, 20]. Regarding its crystallographic structure, the larger lobe of the C-terminal is comprised a substrate-binding site made up of α-helices, whilst the N-terminal is comprised an ATP binding pocket and is predominantly made up of β-strands [20]. SRPK1 activity is governed by its sub cellular location and the level of dephosphorylation of its substrate [21, 22]. The elimination of the SRPK1 spacer domain aggregates splicing factors, leading to alterations in gene expression. The cytoplasmic attachment of SRPK1 is facilitated by its interaction with heat shock protein Hsp40 and molecular chaperone Aha1 [23]. This interaction between molecular chaperones and SRPK1 results in translocation of the kinase.

SRPK1 is known to be overexpressed in numerous malignancies and has been implicated in various oncogenic signalling pathways across a multitude of cancer types [16, 17, 24–49].The aim of this review is to systematically summarise all the studies published to date which examine the relationship between SRPK1 expression and cancer development and prognosis. Where available we have highlighted specific aberrant signalling pathways through which SRPK1 has been found to promote oncogenesis.

## Materials and methods

### Literature search and study selection

This systematic review adhered to the recommendations of the PRISMA (Preferred Reporting Items of Systematic Reviews and Meta-analysis) statement [50]. A systematic search of PubMed, Embase, and the Cochrane Central Register of Controlled Trials was performed for all studies that investigated the role of SRPK1 in cancer pathogenesis. The following search terms were used in the search algorithm: (Serine-Arginine Protein Kinase 1 OR SRPK1) AND (cancer OR adenocarcinoma). A second search strategy was used to identify manuscripts detailing the role of SRPK1 in chemotherapy response: (Serine-Arginine Protein Kinase 1 OR SRPK1) AND (chemotherapy). The latest search was performed on the first of September 2021. Two authors (W.P.D and E.O’C.) independently examined the title and abstract of citations, and the full texts of potentially eligible studies were obtained; disagreements were resolved by discussion. The reference lists of all articles that were retrieved were further screened for additional eligible publications.

### Eligibility criteria

All studies that investigated the prognostic role of SRPK1 in cancer or the mechanisms or pathways by which SRPK1 impacted a distinct oncogenic process or response to chemotherapy, were deemed eligible for inclusion. This included studies which evaluated patient samples, animal models, cell lines and publicly available genomic databases. Review articles and articles relating to SRPK1s role in other biological or pathological processes, not pertaining to cancer were not deemed eligible. Studies which explored the structural or organic properties of SRPK1 inhibitors, without inclusion of in vivo/in vitro experiment component were also excluded. There were no language restrictions.

### Analysis

The results of all eligible studies were grouped together by the organ involved. The impact of SRPK1 expression on prognosis was described where available (Table 1). The association between SRPK1 and apoptosis, cell growth, invasion, migration, and treatment response in vitro as well as tumour growth, tumourigenicity, metastasis and treatment response in vivo were described. Where available, the signalling pathways involved are also discussed (Table 1).

**Table 1:** Summary of the signalling pathway involvement and prognostic role of SRPK1 in various cancers

Cancer type (primary location)	Signalling pathway		Prognostic implication	References	
Basal cell carcinoma	SOX2↑ SRPK1↑ PI3K/AKT↑		–	Li et al. [81]	
Breast cancer	–		Increased SRPK1 expression was associated with poor disease outcomes regardless of ER status	Van Roosmalen et al. [24]	
	–		Increased SRPK1 expression correlated with TNM stage and prognosis	Li et al. [52]	
	SRPK1↑ AKT/MAPK↑		Increased SRPK1 expression correlated with tumour grade	Hayes et al. [25]	
	SRPK1↑ RBM4 maintained in cytoplasm, promoting splicing of anti-apoptotic MCL-1 isoform		SRPK1 expression found to be elevated in breast cancer tissue	Lin et al. [26]	
	LIMK2↑ SRPK1↑ NF-KB signalling↑		–	Malvi et al. [51]	
	MiR 9↓ SRPK1↑ Promotion of metastasis in triple negative breast cancer↑		–	Selcuklu et al. [54]	
Colorectal cancer	–		SRPK1 expression was found to be elevated in colorectal cancer tissue. Expression level correlated positively with LN metastasis and disease stage	Yi et al. [61]	
	SRPK1↑ AKT/MAPK↑		Increased SRPK1 expression correlated with tumor grade in colon cancer	Hayes et al. [25]	
	SRPK1↑ PHLPP↓ AKT Phosphorylation↑		Elevated SRPK1 and AKT expression were found in colon cancer tissue	Wang et al. [27]	
	SRPK1↑ PP1a↓ Preferential splicing of MKNK2B↑		Colorectal cancer specimens were found to have higher expression of SRPK1, which correlated with large tumor size and advance stage disease	Liu et al. [28]	
	MiR 216b↓ SRPK1↑		SRPK1 expression is elevated in colorectal cancer tissue and correlates with TNM stage and LN metastasis	Yao et al. [29]	
	SRPK1↑Cleaved PARP↑BCLX↑NFKb↑		SRPK1 expression was found to be elevated in colorectal cancer, a correlation was noted between expression level, TNM stage and prognosis	Huang et al. [30]	
	MALAT 1↑ SRPK1↑ AKAP9↑		–	Yang et al. [62]	
	SRPK1/WNK1/GSK3↑ SLC39A14 oncogenic isoform ↑		–	Thorsen et al. [56]	
Endometrial cancer	–		SRPK1 expression was found to be elevated in endometrial cancer tissue compared to normal endometrium and was associated with poor prognosis	Kurimchak et al. [31]	
Esophageal cancer	SRPK1↑ AKT↑ JNK↓ TGF-beta↑		SRPK1 expression was found to be elevated in Esophageal SCC,and correlated with poor prognosis and risk of metastasis	Ren et al. [32]	
Gastric cancer	IGF-1↑ SRPK1↑ EMT markers↑		SRPK1 expression was elevated in gastric cancer compared to match normal gastric tissue. SRPK1 expression correlated with grade and stage of disease as well as LN metastasis	Wang et al. [33]	
	–		SRPK1 expression was found to be elevated in gastric cancer and was a predictor of TNM stage and poor prognosis	Xu et al. [34]	
	–		Elevated SRPK1 expression was associated with gastric cancer compared to matched normal tissue	Li et al. [36]	
	MiR 126↓ SRPK1↑		SRPK1 expression was elevated in gastric cancer tissue compared to matched normal gastric tissue, a correlation was noted between SRPK1 expression LN metastasis and poor prognosis	Li et al. [35]	
Glioma	–		SRPK1 expression was elevated in glioblastoma tissue samples and was associated with poor survival	Sigala et al. [17]	
	–		SRPK1 expression was elevated in Glioma tissue and rarely expressed in normal adjacent tissue. SRPK1 expression had a greater association with low grade rather than high grade glioma	Wu et al. [37]	
	SRPK1↑ BCL2↑ BAX↓ AKT/E1F4E phosphorylation↑		–	Chang et al. [67]	
	Plexin B1↑ SRPK1↑ PI3K/AKT↑		–	Chang et al. [68]	
HCC	–		SRPK1 expression was upregulated in HCC tissue samples, and demonstrated a correlation with disease stage, survival and gender	Zhang et al. [38]	
	SRPK1↑ PI3K/AKT↑		SRPK1 expression was elevated in HCC tissue compared to matched normal hepatic tissue	Zhou et al. [37]	
	SRPK1↑ CHK1-S↑		SRPK1 mRNA expression was elevated in HCC compared to matched normal hepatic tissue	Hu et al. [39]	
	(Hypoxia)MiR 1296↓ SRPK1↑ AKT phosphorylation↑		Elevated SRPK1 and downregulated MiR- 1296 expression are associated with adverse clinical features and poor prognosis in HCC patients	Xu et al. [40]	
	MiR 155↓ SRPK1↑		–	Wang et al. [69]	
AML	SRPK1↑BRD4 long isoform↑ BCL2,MYC↑		–	Tzelepis et al. [71]	
CML	SRPK1↑ PARP,BAX and Caspase 3↓ BCL2↑		–	Wang et al. [72]	
CML	WT1↑Basp1↓ SRPK1↑		–	Belali et al. [73]	
T-ALL	SRPK1↑ PI3K/AKT↑		–	Siqueira et al. [74]	
Lung cancer	–		Elevated SRPK1 expression was found in both lung adenocarcinoma and SCC compared to normal lung parenchyma	Gout et al. [41]	
	–		SRPK1 expression was found to be upregulated at both a protein and mRNA level in NSCLC	Liu et al. [42]	
	SRPK1↑GSK3-beta phosphorylation↑ β catenin/TCF signalling ↑		SRPK1 expression was elevated in NSCLC tissue compared to normal matched tissue, correlating strongly with TNM stage and poor prognosis	Gong et al. [43]	
	FGF 2↑ SRPK1↑ VEGFR1 pro-angiogenic isoform↑		–	Jia et al. [76]	
Melanoma	SRPK1↑ Pro-angiogenic VEGF isoform↑		–	Gammons et al. [79]	
Ovarian cancer	–		SRPK1 expression, was more commonly elevated in ovarian cancer tissue compared to normal ovarian tissue	Odunsi et al. [44]	
	UCA1↑ SRPK1↑		SRPK1 was found to have higher expression in ovarian cancer tissue compared to normal ovarian tissue	Wang et al. [45]	
Prostate cancer	SRPK1↑ Pro-angiogenic VEGF isoform↑		SRPK1 expression was elevated in malignant prostate tissue and PIN compared to benign prostatic tissue	Mavrou et al. [46]	
	–		SRPK1 expression was elevated in prostate cancer tissue compared to benign prostatic tissue. SRPK1 expression correlated with pT stage, extracapsular invasion, perineural invasion but not gleason grade	Bullock et al. [47]	
	–		SRPK1 expression was elevated in prostate cancer tissue, correlating with worse overall survival and Prostate cancer-specific mortality	Abou-Ouf et al. [48]	
	WT1↑Basp1↓ SRPK1↑		–	Belali et al. [73]	
RCC	SRPK1↑ PI3K/AKT↑		–	Han et al. [78]	
Retinoblastoma	–		SRPK1 underexpression is associated with advanced, large retinoblastoma tumours	Krishnakumar et al. [49]	
Testicular cancer	–		SRPK1 expression in GCTs was elevated, to similar levels as to what is seen in normal testicular tissue. Downregulation of SRPK1 is associated with a poor prognosis and cisplatin resistance	Schenk et al. [16]	

## Results

### Literature review

**Fig. 1:**
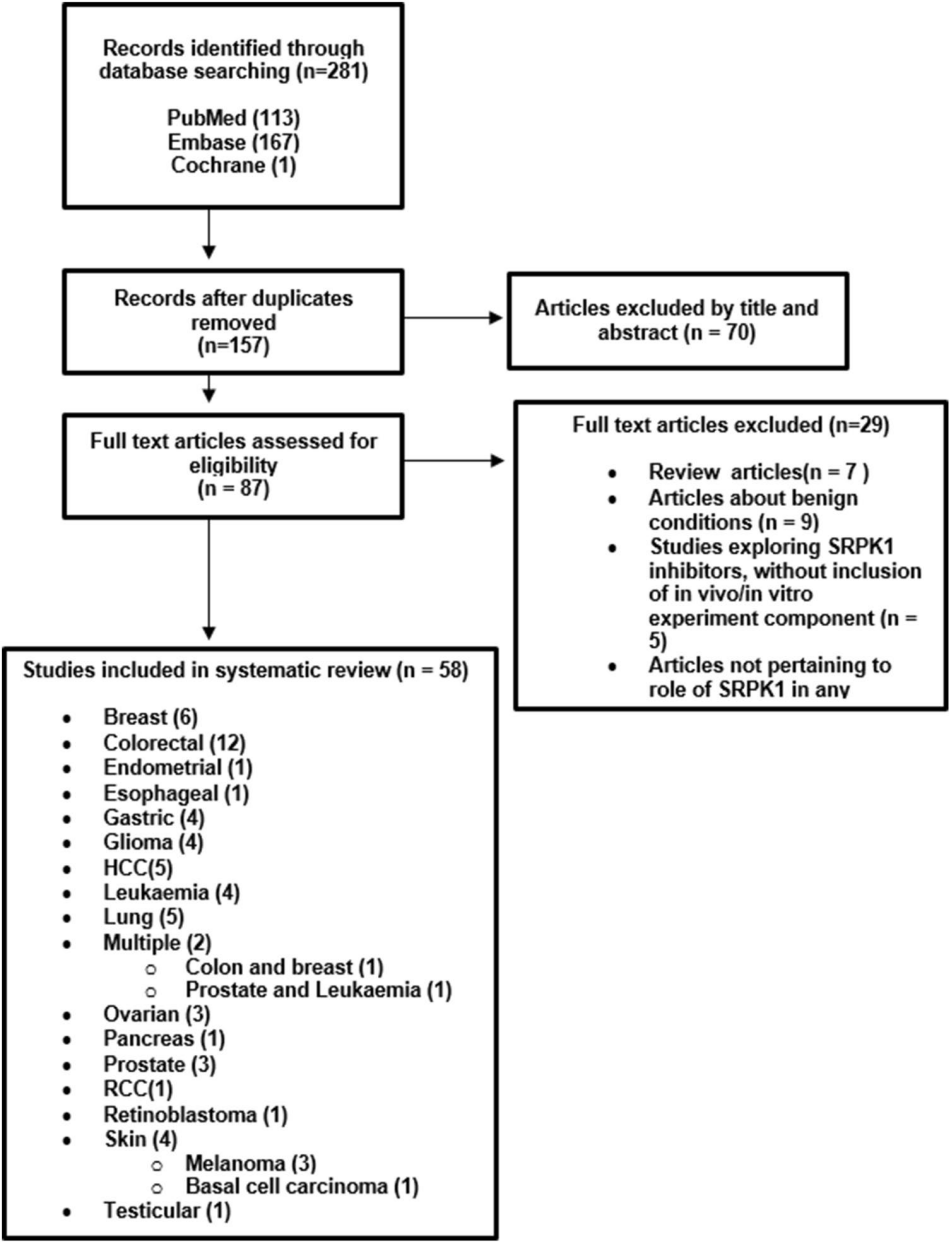
PRISMA 2020 flow diagram

The initial search yielded 281 publications; this was reduced to 157 after duplicates were removed with a further 70 papers excluded by title and abstract alone, leaving 87 manuscripts for full-text review. 29 articles were deemed ineligible after full-text review and the remaining 58 articles were deemed suitable for inclusion in the systematic review. Of note, two of these articles investigated the role of SRPK1 in more than one cancer type. The reasons as to why the articles were excluded are listed in the PRISMA flow diagram (Fig. 1).

### Breast cancer

We identified seven papers which investigated the role of SRPK1 in breast cancer [24–26, 51–54]. SRPK1 expression is higher in breast cancer tissue compared to matched normal tissue, where expression is confined largely to ductal epithelium [25]. SRPK1 silencing results in increased rates of apoptosis, and decreased phosphorylation of mitogen-activated protein kinase 3 (MAPK3), MAPK1 and protein kinase B (AKT) in breast cancer cell lines, suggesting a likely relationship between SRPK1 and AKT/MAPK signalling pathways [25]. SRPK1 and the splicing factor RNA-binding protein 4 (RBM4) are overexpressed in breast cancer tissue. SRPK1 maintains RBM4 in the cytoplasm of breast cancer cells promoting preferential splicing of the anti-apoptotic myeloid leukaemia 1 (MCL-1) long isoform [26].

Knockdown of SRPK1 reduces migratory capacity in estrogen receptor negative breast cancer cells. SRPK1 was found to be involved in nuclear factor kappa-light-chain-enhancer of activated B cells (NF-κB) signalling and its silencing was found to impact both canonical and non-canonical pathways in vitro, and metastatic spread to both lung and brain in vivo, but interestingly not liver or bone. Unexpectedly, SRPK1 expression was not found to be associated with AS in this study [24].

Stable isotope labelling by amino acids in cell culture (SILAC) analysis has identified SRPK1 as a protein with a downstream response to LIM domain kinase 2 (LIMK2) inhibition. LIMK2 expression is associated with metastatic spread in triple negative breast cancer. Pharmacological inhibition of SRPK1 in triple negative breast cancer cell lines results in a reduced capacity for invasion and migration, supporting a link between SRPK1 and LIMK2 signalling in the context of metastatic spread in triple negative breast cancer [51].

Tip60 acetylation of SRPK1 is a key step in the sensitisation of breast cancer cells to cisplatin. Tip60 acetylation was found to destabilise SRPK1, impeding its nuclear transport, which resulted in a lower half-maximal inhibitory concentration (IC50) in MCF and 231 cell lines in response to cisplatin treatment [53]. Micro RNA-9 (miRNA-9) is under-expressed in breast cancer cell lines, its over-expression is associated with reduced cell invasion, increased apoptosis and reduced proliferation; miRNA-9 is thought to likely carry out its function by regulating SRPK1 activity [54].

### Colorectal cancer

Thirteen studies explored the role of SRPK1 in colorectal cancer [3, 25, 27–30, 55–61]. SRPK1 expression was generally found to be elevated in colorectal cancer, with the exception of the mucinous subtype [3, 25, 27–30, 60, 61] (Table 1). SRPK1 silencing was found to inhibit proliferation, migration and invasion and increase rates of apoptosis of colorectal cancer cells across a number of included studies [25, 30, 61].

**Fig. 2:**
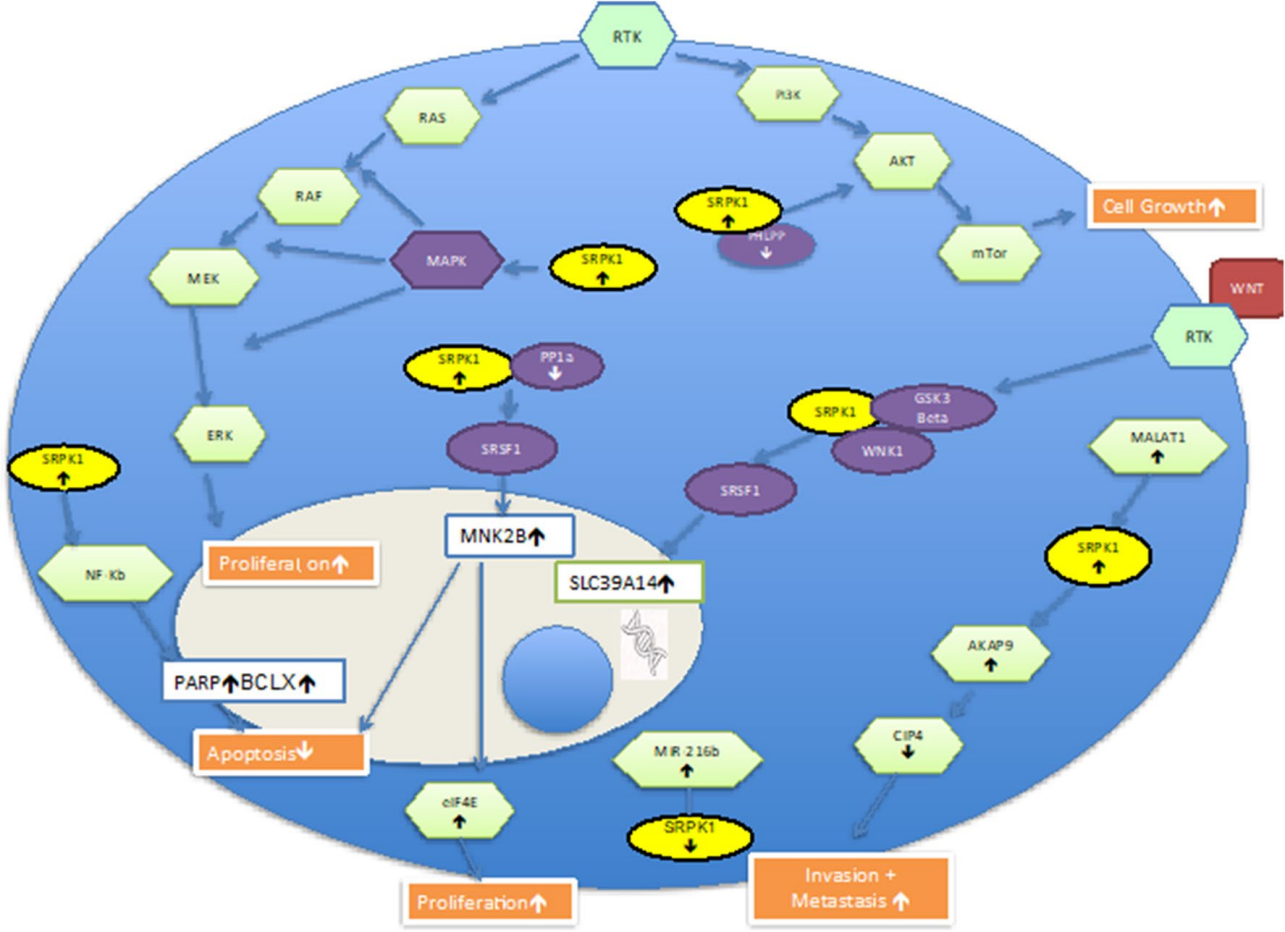
The multimodal involvement of SRPK1 within various oncogenic signalling pathways in an individual tumour type are exhibited here through the example of a colorectal cancer cell

SRPK1 is implicated in a host of signalling pathways known to drive oncogenesis in colorectal cancer (Fig. 2). Similar to what was observed in breast cancer cells, silencing of SRPK1 was found to inhibit MAPK/AKT signalling in colonic cancer cells [25]. An antagonistic relationship was found to exist between SRPK1 and PH domain and Leucine rich repeat protein phosphatases (PHLPP) in controlling AKT phosphorylation in colonic cells, interestingly both under and overexpression of SRPK1 were found to induce constitutive AKT activation in this study [27].

MAP kinase-interacting serine/threonine-protein kinase 2B (MKNK2B) is known to exert a distinct oncogenic effect through MAPK signalling and phosphorylation of eukaryotic translation initiation factor 4E (E1F4E). In contrast the MKNK2A isoform has a pro-apoptotic function. Liu et al.found elevated SRPK1 expression to be associated with preferential MKNK2B splicing in colon cancer cells. Serine/theorine-protein phosphatase (PP1a) was found to have an antagonistic effect favouring MKNK2A splicing in this study [28]. Elevated A-kinase anchor protein 9 (AKAP9) expression has previously been found to enhance tumour growth and metastasis in vivo [55]. Yang et al.identified the long non-coding RNA MALAT1 as having a key role in promoting AKAP9 expression in colon cancer cells via phosphorylation of the SRPK1/ serine-arginine splicing factor 1 (SRSF1) axis [62].

SRPK1 activation is associated with splicing of the BRAF and serrated polyp morphology associated, ras-related C3 botulinum toxin substrate 1B (RAC1B) isoform [57, 63]. Interestingly formation of a lysine deficient protein kinase 1 (WNK1)/glycogen synthase kinase 3 beta (GSK3B) /SRPK1 complex, was found to be necessary to incur SRSF1 phosphorylation and RAC1B splicing in this study. Ibuprofen treatment disrupts this complex in vitro [58]. This may provide a further pharmacological explanation as to the mechanism by which cyclo-oxygenase (COX) inhibition prevents gastrointestinal polyp formation [64, 65]. AS of the cadmium transporter SLC39A14 is associated with colorectal adenoma and carcinoma development. SRPK1 expression is responsible for preferential splicing of its oncogenic isoform in colorectal cancer cells, and its expression is regulated via the wingless/integrated (Wnt) signalling pathway [56]. miRNA-216b targets the 3’UTR of SRPK1 directly, and suppresses proliferation, migration and invasion in colorectal cancer cells, through SRPK1 inactivation [29].

Huang et al. explored the relationship between elevated SRPK1 expression, apoptosis inhibition and oxaliplatin resistance in colorectal cancer cells. SRPK1 silencing was found to promote cleaved poly (ADP-ribose) polymerase (PARP) and b-cell lymphoma extra S (BCL-xS) expression in cancer cells. NF-κB signalling was also found to be downregulated in response to SRPK1 silencing and this was associated with a significant reduction in oxaliplatin IC50 values on MTT assay [30]. Plascencia et al., also previously provided evidence linking SRPK1 expression to oxaliplatin resistance in colorectal cancer [59]. Interestingly, interrogation of the cancer genome atlas (TCGA), found SRPK1 expression to be significantly lower in mucinous colon tumours compared to non-mucinous, with reduced expression correlating with reduced survival [3, 60]. This is potentially pertinent given the poor response of this distinct molecular subtype to standard adjuvant treatment regimens [66]

### Endometrial cancer

Using multiplexed inhibitory beads and mass spectrometry, the kinome profile of primary endometrial tumours was analysed in detail. SRPK1 was identified as having a likely role in primary endometrial cancer development. Pharmacological inhibition of SRPK1 with SPHINX31 was found to inhibit cell proliferation and induce apoptosis in endometrial cancer cells. Interestingly activation of epidermal growth factor receptor (EGFR)/insulin-like growth factor 1 receptor(IGFR-1)/AKT signalling, a pathway previously shown to be associated with elevated SRPK1 expression, promoted resistance to SRPK1 inhibition, suggesting a potential feedback loop mechanism in endometrial cancer cells [31].

### Esophogeal cancer

Elevated SRPK1 protein expression is associated with esophageal squamous cell carcinoma (SCC). SRPK1 silencing inhibits proliferation, invasiveness and migration and induces apoptosis across esophageal SCC cell lines. Further analysis demonstrated a decrease in phosphorylation of AKT and increase in phosphorylation of Jun N-terminal kinase (JNK) in response to SRPK1 silencing, indicating a key role of SRPK1 in mediating transforming growth factor beta (TGF-B)-induced proliferation and apoptosis in this context [32].

### Gastric cancer

Four studies were identified which examined the role of SRPK1 in gastric cancer development [33–36]. All included studies found SRPK1 to be overexpressed in gastric cancer tissue compared to matched normal tissue (Table 1). Wang et al.found SRPK1 silencing to inhibit cell cycle progression, migration and invasion in gastric cancer cells. Elevated SRPK1 expression was found to be associated with increased levels of IGF-1 as well as levels of epithelial-mesenchymal transition (EMT) biomarkers; N-cadherin, matrix metalloproteinase 2 (MMP2) and zinc finger protein SNAI2 (Slug) [33].

Protein phosphatase 2 (PP2A) and dual-specificity phosphatase (DUSP) expression were found to alleviate the oncogenic effects of SRPK1 expression in gastric cancer cells, though their exact inhibitory roles were not fully determined in this study [34]. SRPK1 knockdown was found to suppress gastric cancer cell proliferation and tumour growth, both in vitro and in vivo. DNA microarray analysis identified a potential link between SRPK1 expression and the proliferation of a number of small nucleolar RNA (SnoRna), including SnoRnaD10, SnoRnaA42 and SnoRnaA74A all of which have been linked to gastric cancer progression [36].

Li et al.identified a potential relationship between SRPK1 and miRNA-126. IHC analysis found an antagonistic relationship to exist between SRPK1 and miRNA-126 expression, whereby miRNA-126 is underexpressed and SRPK1 overexpressed in gastric cancer tissue. This finding correlated with lymph node metastasis and poor prognosis in patient samples. miRNA-126 expression was found to inhibit proliferation, migration and invasiveness of gastric cancer cells. A dual luciferase reporter assay was performed which confirmed SRPK1 as a specific target gene of miRNA-126 [35].

### Glioma

Four studies examined the role of SRPK1 in glioma development [17, 37, 67, 68]. Interestingly aberrant SRPK1 expression was consistently found in glioma tissue samples and cell lines, though its expression has scarcely been found in normal glial cells [17, 37]. Sigala et al.found RNA silencing of SRPK1 to have little impact on cell viability in vitro, though it was found to induce cisplatin sensitivity [17]. In contrast, Wu et al.found SRPK1 knockdown of glioma cells to inhibit growth, migration and invasion capacity in normoxic and to a degree in hypoxic conditions. Most notably, however, SRPK1 knockdown was associated with cisplatin resistance in this study [37].

Chang et al*.* found SRPK1 knockdown to be associated with cell apoptosis, decreased migration and invasion in vitro and to significantly reduce tumour growth in vivo. SRPK1 silencing had a significant impact on cell apoptosis via Bcl-2 down regulation and Bax activation. AKT /E1F4E phosphorylation were also inhibited by SRPK1 silencing, as were hypoxia-inducible factor 1 (HIF-1) and VEGF production [67]. The same group later found Plexin B1 also to be overexpressed in glioma cell lines. Plexin B1 was found to promote SRPK1 activity via PI3K/AKT signalling, resulting in an increase in cell growth, angiogenesis and motility, both in vitro and in vivo [68].

### Hepatocellular carcinoma (HCC)

Five studies evaluated the role of SRPK1 in hepatocellular carcinoma (HCC) [37–40, 69]. SRPK1 expression is elevated in HCC [37–40]. SRPK1 knockdown was associated with decreased cell proliferation and reduced tumour growth in vivo [37]. Western blot analysis revealed an association between SRPK1 expression and the PI3K/AKT signalling pathway [37]. Aberrant SRPK1 expression is associated with AS of the checkpoint kinase 1 short (CHK1-S) isoform, which is highly expressed in HCC and associated with poor prognosis [39].

Two studies examined potential relationships between SRPK1 and miRNA in HCC development [40, 69]. miRNA-1296 is under-expressed in HCC tissue and cells, it interacts directly with SRPK1, likely regulating its function in normoxic conditions [40]. Hypoxia was found to play a key role in inhibiting miRNA-1296 expression, resulting in an SRPK1/AKT mediated increase in migration and invasion in HCCLM3 cell lines in vitro [40]. Similarly, miRNA-155 was found to be under-expressed in HCC tissue compared to normal hepatic tissue, its up-regulation inhibited proliferation, migration and invasion in HCC cells [69].

### Leukaemia

Five studies examined the role of SRPK1 in the development of various luekaemias [70–74]. Siqueira et al., found SRPK1 to be overexpressed in myeloid and lymphoid leukaemia cell lines. Pharmacological inhibition with SRPIN340 demonstrated a cytotoxic effect, impacting expression of VEGF, fas cel surface death receptor (FAS), MAPK2K1 and MAPK2K2 [70]. Tzelepis et al*.* found SRPK1 knockdown to elicit increased acute myeloid leukaemia (AML) cell apoptosis in vitro and in vivo. Pharmacological SRPK1 inhibition with SPHINX31, was found to result in AS of bromodomain-containing protein 4 (BRD4) towards its long isoform. Notably this isoform unlike the BRD4 short isoform, is not associated with enhanced expression of BCL2 and MYC [71].

SRPK1 silencing is associated with a significant increase in apoptosis in K562 chronic myeloid leukaemia cells. Western blot analysis showed an increase in expression of PARP, BAX and Caspase 3 as well as a reduction in BCL2 expression, in response to SRPK1 silencing [72]. Wilms tumour 1 (WTI) expression is associated with increased SRPK1 expression in K562 cells, meanwhile brain abundant membrane attached signal protein 1 (BASP1) demonstrated an antagonistic effect in controlling SRPK1 activity in this study [73].

SRPK1 expression is associated with PI3K/AKT signalling in T-cell acute lymphoblastic leukaemia (T-ALL) cell lines. Interestingly SRPK1 inhibition alone was not found to effect cell apoptosis in this study, rather synergistic treatment alongside an AKT inhibitor was found to have a significant effect on apoptosis. This suggests the likely presence of a regulatory feedback loop within the signalling cascade in this cell type [74].

### Lung cancer

Five studies explored the role of SRPK1 in Lung cancer [41–43, 75, 76]. SRPK1 expression is elevated in Non Small Cell Lung Cancer (NSCLC) tissue and is associated with increased growth and migration in NSCLC cells [41–43]. SRPK1 expression activates beta-catenin/TCF signalling via phosphorylation of GSK3-beta [42]. Increased beta-catenin signalling results in a cancer stem cell phenotype in NSCLC [43]. Inhibition of this signalling pathway via introduction of a chimeric antibody targeting SRPK1 activity has been found to inhibit cell growth, migration and invasion in vitro and tumour growth in vivo [75].

A recently published article identified a further role for SRPK1 in NSCLC development. Fibroblast growth factor -2 (FGF-2) was found to activate SRPK1 amongst other splicing proteins to promote VEGFR1 AS in NSCLC cells, contributing to angiogenesis and progression of NSCLC [76].

### Ovarian cancer

Three studies explored the role of SRPK1 in ovarian cancer [44, 45, 77]. Two of these studies found SRPK1 expression to be upregulated in ovarian cancer tissue [44, 45] (Table 1). SRPK1 silencing was found to inhibit cell proliferation and enhance cisplatin sensitivity in SKOV3 cells [44]. Wang et al.found the long non-coding RNA UCA1 to be overexpressed in ovarian cancer. Overexpression of UCA1 was found to be associated with enhanced migration, invasion and cisplatin resistance in SKOV3 cells. The effects of UCA1 overexpression were found to be partly mitigated by SRPK1 silencing [45]. By contrast to the aforementioned studies, Schenk et al. found SPRK1 overexpression to induce cisplatin sensitivity in the A2780 ovarian cancer cell line [77].

### Pancreatic cancer

A single study explored the role of SRPK1 in pancreatic cancer [15]. SRPK1 expression is elevated in malignant and dysplastic pancreatic tissue compared to normal pancreatic tissue. SRPK1 silencing inhibits proliferation and induces apoptosis in pancreatic cancer cells, and enhances their sensitivity to gemcitabine and cisplatin treatment [15].

### Prostate cancer

Four studies examined the role of SRPK1 in prostate cancer [46–48, 73]. SRPK1 expression is elevated in both malignant prostate cancer and interestingly prostatic intraepithelial neoplasia (PIN) [46–48](Table 1). SRPK1 silencing in prostate cancer cells was found to result in preferential splicing of the anti-angiogenic VEGFA isoform. SRPK1 silencing did not impact cell proliferation, invasion or migration in vitro, but was shown to stunt tumour growth in vivo in this study [46]. Pharmacological inhibition of SRPK1 in PC3 prostate cancer cells, reduces cell proliferation, invasion and migration. WTI expression was found to be associated with increased SRPK1 expression in this study, with BASP1 demonstrating an antagonistic effect in controlling SRPK1 activity [73].

### Renal cell carcinoma (RCC)

One study examined the role of SRPK1 in renal cell carcinoma(RCC) [78]. SRPK1 protein and mRNA expression was found to be elevated in RCC patient samples. SRPK1 silencing inhibits cell proliferation, migration and invasion in vitro and tumourigenesis in vivo, its activity is linked to PI3K/AKT signalling [78].

### Retinoblastoma

A single publication examined the role of SRPK1 in retinoblastoma [49]. Under-expression of SRPK1 is associated with cisplatin resistance and recurrence in this study [49].

### Skin (melanoma and basal cell carcinoma (BCC))

Three studies were identified, which explored the relationship between SRPK1 and melanoma [14, 79, 80]. Gammons et al.found SRPK1 expression to be elevated in both uveal and cutaneous melanoma cell lines. SRPK1 silencing was found to result in AS of the anti-angiogenic VEGF isoform and was associated with inhibition of cell growth in vivo. However, silencing was not found to impact tumour growth in vitro [79]. Moreira et al., found pharmacological inhibition of SRPK1 to inhibit migration and invasion of melanoma cells in vitro, and metastasis in vivo [80].

SRY-box 2 (SOX2) expression is elevated in BCC tumour samples, and its knockdown inhibits migration and invasion of BCC cells in vitro. SOX2 mediates its affect through an interaction with SRPK1 resulting in upregulation of PI3K/AKT signalling [81].

### Testicular germ cell tumors (GCT)

A single study evaluated SRPK1 expression in testicular germ cell tumors (GCTs) [16]. Though SRPK1 is generally found to be highly expressed in these tumours, SRPK1 downregulation correlated positively with cisplatin resistance and poor prognosis in this study [16].

## Discussion

Elevated SRPK1 expression is commonly found in human epithelial cancers and often correlates positively with advanced disease stage and poor survival (Table 1). SRPK1 expression is also elevated in the precursor lesions of some epithelial malignancies, highlighting the enzymes likely role in the early stages of oncogenesis in such cancers [15, 46, 56]. Current available evidence, suggests a likely future role for SRPK1 as a prognostic biomarker in some more common epithelial cancers. Interestingly, however, underexpression of SRPK1 is also associated with a poor outcome in some non-epithelial derived malignancies. Both Schenk et al*.* and Krishnakur et al*.* found downregulation of SRPK1 to be associated with cisplatin resistance and a worse prognosis in testicular GCT’s and retinoblastomas, respectively [16, 49]. At present, little is understood as to how SRPK1 expression is protective in these malignancies.

SRPK1 is implicated in the promotion of each of the hallmarks of cancer across one tumour type or another [24, 25, 30–33]. As such it has become an attractive therapeutic target. Inhibitors such as the SRPK1/2 inhibitor SRPIN340 and the more specific SPHINX and SPHINX31 have been used to good effect in pre-clinical studies (Table 2) [79]. For example SPHINX31 has been shown to induce cell cycle arrest and effect leukaemogenesis in AML, similarly SPHINX was found to promote splicing of the anti-angiogenic VEGF165b isoform in prostate cancer cells and reduce tumour growth in vivo [46, 71]. However the side-effect profile of SRPK1 inhibition has not yet been illustrated. Given the multiple roles played by SRPK1 across various oncogenic processes, its inhibition is likely to impact important normal cellular processes also. Further studies to explore its side-effect profile are warranted.

**Table 2:** Examples of SRPK1 inhibitors

Inhibitor	Target	Developmental stage	Target disease
SRPIN340	SRPK1/2	Preclinical	T-ALL [74] Melanoma [80, 86]
SRPKIN-1	SRPK1/2(Irreversible)	Preclinical	Age-related macular degeneration [8]
SRPIN803	SRPK1/CK2	Preclinical	Age-related macular degeneration [87]
SPHINX	SRPK1	Preclinical	Leukaemia [73] Prostate cancer [73]
SPHINX31	SRPK1	Preclinical	Diabetic retinopathy [88] AML [71]

miRNA-based therapeutics are emerging as an exciting cancer treatment option [82, 83]. Four SRPK1 specific miRNA have been identified, each of which has been found to regulate or suppress SRPK1 activity [29, 35, 40, 69]. It is probable that further studies will reveal miRNA to have a more prominent role in the regulation of SRPK1, with further SRPK1-specific mi-RNA likely to be identified in the context of other cancers. Interestingly miRNA-1296 which is known to regulate SRPK1 activity in HCC, is underexpressed in hypoxic conditions leading to increased SRPK1 activity [40]. Similarly in glioma cells, hypoxic conditions were found to reduce the impact of SRPK1 inhibition on tumour growth, invasion and migration [37]. More analysis regarding the impact of environmental factors on SRPK1 activity are warranted.

Resistance to chemotherapy, remains a main cause of treatment failure and death in cancer patients [84]. SRPK1 silencing has been linked to platinum based chemotherapy sensitisation in breast, colorectal, pancreatic and ovarian cancer [15, 25, 44]. Meanwhile its inhibition has been linked to resistance to the same family of chemotherapy agents in testicular GCTs, retinoblastoma, glioma and ovarian cancer [16, 37, 49, 77]. SRPK1 expression has also been shown to be downregulated in mucinous colorectal cancer, a subtype known to respond poorly to adjuvant chemo and radiotherapy [3, 66]. To date only Wang et al*.* have contributed a hypothesis as to how SRPK1 activity is involved in the metabolism of platinum-based chemotherapy [53]. Further studies are warranted to elucidate the mechanisms involved.

SRPK1 is involved in a diverse array of signalling pathways associated with various cancers (Table 1). A number of these pathways have been found to be present across more than one tumour type. For instance SRPK1 has been implicated in promoting AKT signalling in breast, colorectal, esophageal, endometrial and pancreatic cancer as well as glioma, HCC and T-ALL (Table 1). Similarly, SRPK1 promotes NF-КB signalling in both breast and colorectal cancer and AS of the pro-angiogenic VEGF isoform in melanoma, NSCLC and prostate cancer (Table 1). However even within pathways common to multiple tumour types, subtle differences in signalling have been identified. For example, rather unexpectedly pharmacological inhibition of SRPK1 did not interfere with AKT signalling in endometrial cancer cells, with the authors suggesting a feedback loop may be present within the pathway [31]. A similar finding was illustrated in T-ALL cell lines where combined AKT/SRPK1 inhibition was required to impede AKT/PI3K signalling [74]. In contrast SRPK1 inhibition alone is sufficient to interfere with AKT signalling in many other cancers [25, 32, 37, 67, 68, 70]. Molecular differences can also have a profound impact on SRPK1 activity. Schenk et al*.* and Odunsi et al*.* both explored the relationship between SRPK1 expression and the response of ovarian cancer cells to platinum-based chemotherapy. The groups demonstrated opposing findings, with the less common subtype represented by the A2780 cell line demonstrating chemoresistance in response to SRPK1 knockdown, whilst Odunsi et al. found knockdown of SRPK1 to be associated with chemosensitisation of SKOV3 cells [44, 77]. SRPK1 activity appears to be highly specific and sensitive to variations in tumour biology. Little can be extrapolated from one cancer type to explain its activity in another. Further investigation into the contribution of various factors within the tumour microenvironment towards SRPK1 activity are warranted.

It is likely that SRPK1 may have an even broader impact on oncogenesis that what is currently understood. Emerging evidence from investigations into the prognostic role of cancer stem cells, suggest differing isoforms can have very different implications regarding prognostic outcome. For example differing isoforms of CD44 + stem cells have been found to be associated with opposing prognostic outcomes in colorectal cancer [85]. As one of the key moderators of AS, it is likely SRPK1 has a role in cancer stem cell isoform selection, this represents a further potentially exciting avenue of research relating to the role of SRPK1 in oncogenesis that remains unexplored.

In conclusion, SRPK1 activity is prognostic in many epithelial derived cancers (Table 1). It is associated with various oncogenic processes and signalling pathways that are more often than not unique to the specific cancer under examination (Table 1). There remains a need to establish a deeper understanding of factors that influence SRPK1 expression. For example to date there is limited data regarding how SRPK1 expression is influenced by external factors such as the tumour microenvironment. Further proteomic and transcriptomic analysis and evaluation of large data sets may help provide better understanding of its activity in this context.

## Data Availability

All data are available on Pubmed and/or Embase.
